# Opportunities for nurses aspiring to or undertaking clinical academic development globally: protocol for a scoping review

**DOI:** 10.1136/bmjopen-2023-078562

**Published:** 2024-08-31

**Authors:** Claire Jennings, Gill Norman, Marie Marshall, Michelle Briggs

**Affiliations:** 1 Division of Nursing, Midwifery and Social Work, School of Health Sciences, Faculty of Biology, Medicine and Health, The University of Manchester, Manchester, UK; 2 Royal Manchester Children's Hospital, Manchester University NHS Foundation Trust, Manchester, UK; 3 Newcastle University, Newcastle, UK; 4 Liverpool University Hospitals NHS Foundation Trust, Liverpool, UK; 5 School of Health Sciences, University of Liverpool, Liverpool, UK

**Keywords:** EDUCATION & TRAINING (see Medical Education & Training), Protocols & guidelines, Nurses

## Abstract

**Abstract:**

**Introduction:**

Nurse researchers often lack awareness of how to start a clinical academic research career and often lack clear entry routes. This scoping review aims to identify the range and nature of clinical academic opportunities available for nurses. This will also identify the knowledge gaps and provide the basis for future research.

**Methods and analysis:**

The review will be conducted using scoping review methodology and reported in accordance with Preferred Reporting Items for Systematic Reviews and Meta-Analyses with the Extension for Scoping Reviews guidelines. We will search CINAHL (EBSCO), MEDLINE (Ovid), AMED (Ovid) and ProQuest using a search based on the facet’s ‘nurse’, ‘research’ and ‘clinical academic’. Grey literature and a hand search of the reference lists will be conducted for additional publications meeting the inclusion and exclusion criteria. We will include articles published in English, with a focus on registered nurses interested in undertaking clinical academic development within any healthcare system, with no restrictions regarding the date of publication. Following initial screening of titles and abstracts, relevant full-text papers will be screened for inclusion. Two independent reviewers will participate in an iterative process of screening the literature, paper selection and data extraction. We will use a prespecified form to extract data which will be charted and presented in a tabular form. Samples of data extraction and charting will be checked by a second reviewer. This will support a narrative summary structured around key identified variables agreed on iteratively by the review team.

**Ethics and dissemination:**

No ethical or Health Research Authority approval is required to undertake this scoping review. The findings will inform future research exploring the opportunities supporting nurses aspiring to undertake clinical academic development and be disseminated via presentations at national conferences and publications in peer-reviewed journals.

**Study registration:**

Open Science Framework, DOI:10.17605/OSF.IO/WVDXH.

**Strengths and limitations of this study**

- The scoping review will be undertaken and reported following established guidelines to ensure transparency and reproducibility.
- The identification and synthesis of data will include published articles found on the CINAHL, MEDLINE, AMED and ProQuest databases, grey literature available on relevant organisations and professional bodies, and a hand search of reference lists.
- Due to limited resources, only a proportion of the screening will be undertaken by a second reviewer; this increases the likelihood of bias or error, although this potential will be mitigated using a triage process to ensure a second reviewer considers all records if there is any uncertainty.
- Literature not available in the English language will be excluded; we will attempt to mitigate this issue using additional checks and listing of relevant foreign language studies.

## Introduction

 Clinical academics in nursing, midwifery and allied health professions play a pivotal role in the UK NHS by bringing patient-focused insight to the fore and conducting translational research which offers direct benefits to the quality of patient care.^1^ To develop as a clinical academic, combining clinical and research responsibilities, it is imperative that training opportunities are provided to gain skills across both fields. There are key differences between the nursing workforce and that of midwifery and allied health professionals in terms of size and complexity; nurses have been the largest clinical workforce in the UK since 1948^2^ and undertake a myriad of roles. The last 6 years have seen the publication of focused research strategies for nursing, midwifery and allied health professions separately.^3^,^5^ This has given impetus to the need to explore research led by nurses and the contributions they can make as members of multidisciplinary research teams to drive improvements in care.

In medicine and dentistry, the Integrated Academic Training programme is a well-established method of supporting individuals in gaining research experience as part of their clinical training.^2^ Conversely, while national and international nursing bodies recognise that engaging nurses in research is essential for advancing the leadership of the profession, with great support for clinical academic nursing roles,^6^,^9^ there are no established structures or funding models to support this career pathway.

In 2012, the UK Clinical Research Collaboration Sub-committee for Nurses in Clinical Research,^10^ known as the Finch report, made a number of recommendations for preparing and supporting clinical academic nurses of the future. This aimed to build up not just the capacity of nurses as clinical researchers but also the capability in the nursing workforce to enable highly qualified nurses to compete against other health professionals for clinical research funding.^11^

The UK National Institute for Health and Care Research (NIHR) has recognised that nurses and midwives are under-represented in research, and in 2019 the NIHR Nursing and Midwifery Incubator was established to accelerate capacity building and support the development of a skilled clinical academic research workforce across the nursing and midwifery professions.^3^ The need to encourage early career interest in target disciplines was further demonstrated in 2023, with the NIHR Research for Patient Benefit Programme (RFPB) specifying a call for projects from ‘historically under-represented disciplines and specialisms to enable capacity building in these areas’.^2^ In a recent review of RFPB-funded projects, only 8% were led by nurses and midwives, reflecting a lack of funding awards which consequently impacts career opportunities.

However, despite efforts to highlight clinical academic careers, the number of nurses developing a research profile is disproportionately low relative to all health professionals applying for clinical doctoral fellowships.^12^ Nurses often lack awareness of how to start a clinical academic research career, and clear and well-publicised routes of entry are rare. Consequently, the career structure and guidance about how to navigate combining a clinical nursing role with academic development are not well established.^13^ Meanwhile the many different development opportunities becoming available to support nurses embarking on clinical academic careers, and the absence of an established structure, may cause difficulties for clinical managers when trying to plan their operational service.^14^

A review of the literature has highlighted that, in the USA, clinical academic nurses are widely recognised, particularly through the MAGNET framework.^15^ Within this healthcare system, they are often referred to as nurse scientists and have been employed in clinical settings for many years, with more healthcare organisations hiring them as they are recognised as instrumental in pursuing the American Nurses Credentialing Centre MAGNET designation.^9 16^ Thus, MAGNET hospitals have a strong body of evidence demonstrating the value placed on clinical academic nurses supporting evidence-based practice, linked to improved nursing care and patient outcomes.^9^ However within this literature, there is limited detail on the training opportunities available to develop as a clinical academic nurse or nurse scientist.

Additionally, Australia and New Zealand are promoting the development of clinical academic nursing, but as yet do not have any formal clinical academic pathways and limited infrastructure to support this career route.^8 17^

Outside of the USA, the Finch report^11^ recommended a funding infrastructure to support the development of nursing clinical academics in England along an integrated clinical academic pathway; it was envisaged that this would enable the initiation of a variety of programmes and schemes.^1 9 18^ Our scoping review of grey literature has identified various educational and opportunities available, but as yet, this area remains unexplored formally. Anecdotally, there are examples of significant progress promoting clinical academic opportunities at local levels, reported by members of clinical academic networks, but these are not replicated consistently across the UK; instead, they appear dependent on individual partnerships and collaborations between research-active organisations.^18^

In addition to formal routes, mentoring, coaching and advice can be a necessary step to support professional and personal development and aspiration in early career clinical academics.^13^ This support process can help clinical academics by providing pastoral care and helping them deal with the demands of a clinical academic research career.^13^ However, no related literature review has explored the opportunities enabling nurses to develop as clinical academics.

For these reasons, a scoping review will be conducted to systematically map the research done in this area as well as identify any existing gaps in knowledge.^19^

Scoping reviews have been shown to be an essential tool to summarise all studies, in an accessible document, that could inform future actions.^20 21^ As such, our aim is to conduct a comprehensive scoping review on training opportunities that are accessible to nurses interested in pursuing clinical academic development. This will enable us to identify the key characteristics of, or factors related to, clinical academic development opportunities and identify and analyse knowledge gaps for further research.^22^

### Objective

The objective of this scoping review is to systematically scope the literature to identify the range and nature of clinical academic opportunities that are available or can be accessed by nurses. It will also clarify and map the key concepts and definitions of clinical academic opportunities and development (including their aims and integration into practice) underpinning the support required for nurses undertaking clinical academic development, the metrics reported for implementation and outputs from a clinical academic programme or pathway.

## Methods and analysis

The scoping review will be guided by the methodological frameworks proposed by Arksey and O’Malley^19^ and the Joanna Briggs Institute.^23 24^ Thus, the following five steps will be followed in this scoping review:

1. Identifying the research question.
2. Identifying relevant studies.
3. Selecting eligible studies.
4. Charting the data.
5. Collating and summarising the results.

This review will follow the relevant aspects of the Preferred Reporting Items for Systematic Reviews and Meta-Analyses with the Extension for Scoping Reviews (PRISMA-ScR) to ensure thorough reporting and mapping of the body of literature.^25^

### Identifying the research question

The ‘PCC’ approach uses the definition of the population, concept and context to support a clear and meaningful title, objective and inclusion criteria for a scoping review (table 1).^24^

**Table 1:** PCC framework

Population/types of participants	Concept	Context
Nurse	Clinical academicResearch trainingResearch internshipResearch careerNursing research	Any healthcare system

#### Population/types of participants

This review will consider all articles related to registered nurses. Registration and requirements for this may vary in different countries, so we will consider any nurses considered to be or described as ‘registered’ in the country in which they are working. In the UK, nursing is a profession which is regulated by law, and the Professional Qualifications Bill^26^ sets out that a profession is regulated where there is a legal requirement to have certain qualifications or experience to undertake certain professional activities or use a protected title.^27^

#### Concept

The concept is the aspiration to explore or pursue clinical academic development exploration or pursuit of clinical academia, or identification as a clinical academic. A clinical academic will be defined as engaging concurrently in clinical practice and research or providing clinical and research leadership in the pursuit of innovation, scholarship and provision of excellent evidence-based healthcare.^10^ While clear definitions of aspiring clinical academics or those exploring clinical academics are difficult to operationalise, it is important to include this in the concept to capture responses to and engagement with the early stages of clinical academic support such as workshops or taster programmes. The other element of the concept is the programmes or courses that support clinical academic development and are available to nurses, such as internships, fellowships or doctorates. This element seeks to capture both opportunities which promote aspiration and engagement and early-stage participation which will foster these.

#### Context

The context of this review is any healthcare system, including acute care, primary care, social care, rehabilitation and community settings.

The main research question will be ‘What information is available about the clinical academic opportunities that are available to nurses who are aspiring to or pursuing early clinical academic career research development?’

The research sub-questions are as follows:

- What are the aims and characteristics (type, duration, provider) of these development opportunities?
- What are the concepts of clinical academic development and opportunity described by these clinical academic development opportunities?
- What is the uptake and accessibility of the programmes or training on offer?
- What constitutes success (metrics, output) for nurses in aspiring to or pursuing clinical academic development?
- What support (mentorship, supervision, funding) is available for nurses undertaking the opportunities on offer?
- What are the experiences of those undertaking such development and those that are supporting and supervising on such programmes or training?

### Identifying relevant studies

A systematic search strategy was developed to identify the relevant studies for inclusion in the review with extensive review and testing with both healthcare librarians and expert university librarians. Recognition of the need to develop nurses as clinical academics, along with midwives and allied health professionals, has become prominent in the last 20 years; however, we will not use date limits for the search to ensure inclusion of any earlier seminal work in this field.

The following electronic databases will be searched from database inception.

- CINAHL (EBSCO).
- MEDLINE (Ovid).
- AMED (Ovid).
- ProQuest.

An expert university librarian assisted in the development of the search terms to ensure that all relevant material was identified. Terms were tested for sensitivity to detect known relevant articles and specificity in their retrieval of other articles; several terms were not included because this testing process demonstrated that no additional articles retrieved with their use were relevant. Terms are searched as both keywords in the title and/or abstract and subject headings (eg, MeSH, EMTREE) as appropriate. The full search strategies for each database are provided in the online supplemental material.

We will search for grey literature. This will include searching websites of relevant organisations or professional bodies for any documents related to clinical academic development. Organisations have been identified from a preliminary search of the internet via Google, such as the National Institute of Health and Care Research and Health Education England. Additionally, stakeholders from within the authors’ clinical academic networks will be invited to recommend organisations which are promoting local, regional or national clinical academic development opportunities.

Reference lists of the included studies will be checked for further studies.

### Selection of eligible studies

Title and abstract screening will be guided by the PCC framework.

We will include records which meet the following inclusion criteria:

- Studies including nurses described or considered as registered nurses by the study authors. Depending on the volume of literature identified we plan to limit inclusion to studies where the focus is on nurses or at least 50% of the participants are nurses.
- Studies with a focus on, aspiration to, exploration of or pursuit and undertaking of clinical academic development. This will include studies with a focus on programmes or training aimed at encouraging, supporting or advancing clinical academic careers or development for nurses. This will include studies which consider opportunities for nurses to engage in any form of clinical academic development or opportunity, including those which are very early stage and focused on promoting aspiration, as well as those which seek to respond to existing aspirations. We will include studies which describe the opportunities for clinical academic development at any stage from ‘taster’ sessions of workshops, through internships to more formal (eg) fellowship programmes.
- Studies may take place in any country or countries, but the full text needs to be available in English language (due to the lack of funding for translation services).

We will exclude records with any of the following:

- Text that does not refer to nurses interested in or exploring clinical academic development.
- Studies where the focus is on doctors or dentists. We will also exclude studies where a majority of participants are other healthcare professionals such as midwives or other allied healthcare professionals. We will also exclude studies of students in pre-registration training and studies which consider purely academic opportunities (eg, opportunities to become a non-clinical academic).
- Full-text articles that could not be obtained or full text that is not available in the English language.
- Studies available in abstract only.

All identified records will be uploaded into Endnote online software^28^ for deduplication. Records will then be uploaded to Rayyan software^29^ for screening of both title and abstract, and of full text. This supports blinded screening of a proportion of records and shared decision-making between the reviewers. If full-text records cannot be obtained or are not available in the English language, to the reviewers, we will seek assistance from expert university and healthcare librarians to explore further access to ensure inclusion where able.

Abstracts will be reviewed for relevance to the review question, and where potentially eligible the full text will be obtained. Following initial piloting on a small subset of records by two independent reviewers, one reviewer (CJ) will screen the titles and abstracts and a minimum of 30% of records will be independently screened by the second reviewer. Any records identified as being of uncertain relevance or sharing characteristics with such records will also be reviewed by the second reviewer or a third reviewer (GN). Disagreements will be resolved through consensus and, if necessary, in consultation with a third reviewer (GN). Articles that have been identified for full-text review will be screened by one reviewer (CJ), and second and third reviewers (both blinded) will each independently review 20% of the records. Conflicts will be discussed between all three reviewers, and additional reviewers (MB and MM) will also be consulted in all cases of uncertainty. This ‘triage’ process will ensure that all records for which there is any uncertainty over relevance will be double screened as a minimum while being a pragmatic response to limited resources and an anticipated large number of records.

Records excluded at full text will be listed in the review with reasons for exclusion to ensure transparency and rigour. The screening process will be documented in a PRISMA flow diagram.

### Charting the data

We will develop a bespoke data extraction form in Microsoft Excel based on scoping review guidance.^20^ This data extraction tool will be piloted by two reviewers using a small number of papers to ensure that the data relevant to the research question will be collected fully from the articles identified.

The data extraction form will include details of the author(s), year of publication, population studied, context, concept, methodology used and key findings relevant to nurses interested in or undertaking clinical academic development. A separate tool may be used for policy documents or guidance if appropriate. Extraction will be undertaken by one reviewer (CJ), and a second reviewer (GN) will check a proportion of the data extraction to ensure a standardised process; a second reviewer will also be consulted in any cases of uncertainty. Any disagreements or continuing uncertainty will be resolved through consensus or by consultation with a third reviewer (MM or MB).

We anticipate that there may be a need for iterative development of the data extraction approach. Any amendments to the tool after piloting is complete will be recorded, including the reasons for this, for transparency. The final version of the data extraction tool will be included in the appendix of the scoping review report to ensure reproducibility.

### Collating and summarising the results

The extracted data will be charted to map the key characteristics and then represented in a tabular form and as a narrative summary.

The tables will report the

- Publication/production year and country of origin.
- Programme design and aims/objectives.
- Professional background of nurses who are the focus of such programmes or training.
- Characteristics of clinical academic development, such as
  - Marketing and dissemination of the programme.
  - Application requirements.
  - Selection process.
  - Structure.
  - Duration.
  - Provider.
  - Funding
  - Educational strategies and methods employed within the programme.
  - Assessment within the programme.
- Outcomes or metrics reported, including participant or provider experience.
- Supervision, support and mentorship offered or provided to support those interested in or undertaking clinical academic development.

The narrative summary will be structured by the different types of opportunities available and at whom they are aimed. Additionally, it will focus on different types of formal and informal systems used to support nurses undertaking clinical academic careers. It will also consider the role of support from managers, mentors or coaches.

### Patient and public involvement

We did not undertake any public or patient involvement in developing this protocol because the review maps out evidence intended for research capacity building of the nursing workforce. However, stakeholder involvement and engagement will be invited by sharing the protocol with our clinical academic networks to suggest more possible search terms not already considered and confirm the inclusion and exclusion criteria. Additionally, we will seek advice and signposting to any programmes or training opportunities which they may be aware of within their own local or national networks to support the grey literature search.

## Ethics and dissemination

No ethical or Health Research Authority approval is required to undertake this scoping review. The protocol has been registered with the Open Science Framework^30^ to ensure quality through transparent reporting and to prevent overlapping or duplicate work being undertaken prior to publication of the review findings (https://doi.org/10.17605/OSF.IO/ACNZP).

The scoping review results will be disseminated by publication in a peer-reviewed journal (preprint may be used on submission) and submitted for presentation at a national conference. We may also use professional networks to disseminate the results to a wider audience of nurses, midwives and allied healthcare professionals undertaking clinical academic development.

## supplementary material

10.1136/bmjopen-2023-078562 online supplemental file 1

## References

[1] Council of Deans (2013). Clinical Academic Careers for Nursing, Midwifery and the Allied Health Professions, Council of Deans of Health position statement.

[2] NIHR (2023). Integrated Academic Training.

[3] NHS England (2021). Making Research Matter: Chief Nursing Officer for England’s strategic plan for research.

[4] Scottish Government (2017). Nursing 2030 vision - the chief nursing officer’s long term strategy to shape the future of the nursing workforce.

[5] Welsh Government (2022). Chief Nursing Officer for Wales: Priorities 2022 to 2024.

[6] AACN (2016). Advancing Healthcare Transformation: A New Era for Academic Nursing.

[7] Carrick-Sen D, Moore A, Davidson P (2019). International perspectivesof nurses, midwives and allied health professionals clinical academic roles: are we at tipping point?. IJPBLHSC.

[8] McKillop A, Doughty L, Atherfold C (2016). Reaching their potential: perceived impact of a collaborative academic–clinical partnership programme for early career nurses in New Zealand. Nurse Educ Today.

[9] Paterson C, Strickland K (2023). The experiences of clinical academic nurses: a meta-aggregation. Semin Oncol Nurs.

[10] Department of Health (2012). Developing the Role of the Clinical Academic Researcher in Nursing, Midwifery and Allied Health Professions.

[11] Butterworth T, Jackson C (2006). Developing the best research professionals. qualified graduate nurses: recommendations for preparing and supporting clinical academic nurses of the future. Report of the UKCRC Sub Committee for Nurses in Clinical Research (Workforce).

[12] Gibson JME (2019). Shouldn’t we all be clinical academics?. J Adv Nurs.

[13] Baltruks D, Callaghan P (2018). Nursing, Midwifery and Allied Health Clinical Academic Research Careers in the UK.

[14] Newington L, Wells M, Adonis A (2021). A qualitative systematic review and thematic synthesis exploring the impacts of clinical academic activity by healthcare professionals outside medicine. BMC Health Serv Res.

[15] Anderson VL, Johnston ANB, Massey D (2018). Impact of MAGNET hospital designation on nursing culture: an integrative review. Contemp Nurse.

[16] Aspinall C, Slark J, Parr J (2024). The role of healthcare leaders in implementing equitable clinical academic pathways for nurses: An integrative review. J Adv Nurs.

[17] Campbell M, Taylor JR (2000). Academic and clinical collaboration. Contemp Nurse.

[18] Henshall C, Kozlowska O, Walthall H (2021). Interventions and strategies aimed at clinical academic pathway development for nurses in the United Kingdom: A systematised review of the literature. J Clin Nurs.

[19] Arksey H, O’Malley L (2005). Scoping studies: towards a methodological framework. Int J Soc Res Methodol.

[20] Dobrowolska B, Chruściel P, Pilewska-Kozak A (2021). Doctoral programmes in the nursing discipline: a scoping review. BMC Nurs.

[21] Vassie C, Smith S, Leedham-Green K (2020). Factors impacting on retention, success and equitable participation in clinical academic careers: a scoping review and meta-thematic synthesis. BMJ Open.

[22] Munn Z, Peters MDJ, Stern C (2018). Systematic review or scoping review? Guidance for authors when choosing between a systematic or scoping review approach. BMC Med Res Methodol.

[23] Peters MDJ, Marnie C, Colquhoun H (2021). Scoping reviews: reinforcing and advancing the methodology and application. Syst Rev.

[24] Peters MDJ, Marnie C, Tricco AC (2020). Updated methodological guidance for the conduct of scoping reviews. JBI Evid Synth.

[25] Khalil H, Peters MDJ, Tricco AC (2021). Conducting high quality scoping reviews-challenges and solutions. J Clin Epidemiol.

[26] UK Parliament (2022). Professional Qualifications Bill.

[1] Department for Business Energy & Industrial Strategy (2021). UK Regulated Professions and Their Regulators: GOV.UK. www.gov.uk.

[28] Clarivate (2023). Endnote.

[29] Ouzzani M, Hammady H, Fedorowicz Z (2016). Rayyan-a web and mobile app for systematic reviews. Syst Rev.

[30] OSF (2011). Open Science Framework.

