# Supplementary material for: Opportunities for nurses aspiring to or undertaking clinical academic development globally: protocol for a scoping review
Source: BMJ Open. 2024 Aug 31;14(8):e078562. doi: 10.1136/bmjopen-2023-078562 (PMC11367388; doi:10.1136/bmjopen-2023-078562)
Supplement: Supplementary file 2 [file bmjopen-2023-078562.reviewer_comments.pdf]

## PEER REVIEW HISTORY

BMJ Open publishes all reviews undertaken for accepted manuscripts. Reviewers are asked to complete a checklist review form and are provided with free text boxes to elaborate on their assessment. These free text comments are reproduced below.

## ARTICLE DETAILS

| | |
|---|---|
| **TITLE (PROVISIONAL)** | Opportunities for nurses aspiring to or undertaking clinical academic development globally: protocol for a scoping review |
| **AUTHORS** | Jennings, Claire; Norman, Gill; Marshall, Marie; Briggs, Michelle |

## VERSION 1 - REVIEW

| | |
|---|---|
| **REVIEWER NAME** | ***Qirong Chen*** |
| **REVIEWER AFFILIATION** | Central South University, Xiangya School of Nursing |
| **REVIEWER CONFLICT OF INTEREST** | No competing interest. |
| **DATE REVIEW RETURNED** | 22-Sep-2023 |

| | |
|---|---|
| **GENERAL COMMENTS** | General Comment:<br>Thank you for inviting me to review this protocol. This topic is important. However, there are some important problems requiring revisions.<br><br>Specific Comments:<br>TITLE:<br>1. The word "globally" in the title is not appropriate for the following reasons: the database only includes a few English databases; the literature is limited to English-language sources; and the "context" section restricts its coverage to high-income countries.<br>2. The term "mechanism" is not appropriate for the following reasons: based on the specific research question posed in the article, this review only focuses on relevant supporting programs and does not consider other supporting opportunities; it exclusively concentrates on "WHAT-programs" without addressing "HOW-how do these programs support clinical nurses' clinical academic development." Maybe “supporting programs” is more appropriate based on research questions you proposed. If you want to use “supporting programs” in the title, you need to define it clearly and make a summarized initial literature review on “supporting programs” in Introduction. Furthermore, you also need to use consist term in the whole paper, for examples, title, abstract, intro, method…… However, I found you also used “mechanism”, “supporting opportunities”, et al. I think these words have different scopes and definitions; this would make readers confused. KEEP CONSISTENTLY, This is important!<br><br>ABSTRACT:<br>1. “We will include articles with a focus on nurses who are interested in undertaking clinical academic development, or a focus on programmes aimed at supporting clinical academic development for nurses.” The sentence uses 'or' as a connecting word. This means that if the literature solely focuses on 'nurses who are interested in undertaking clinical academic development,' it could be included. However, it just means that the population criterion has been met. |

| | |
|---|---|
| | Nevertheless, I believe that the included literature should meet all the criteria of PCC.<br>2. “A proportion…” is not appropriate. The reason will be shown in the comments for Methods.<br><br>STRENGTHS AND LIMITATIONS:<br>1. BMJ Open requires authors to write strengths and limitations of METHODS. The first one and the fourth on could not meet the criterion.<br><br>INTRODUCTION:<br>1. I would like to see a clearer and longer explication of what is already known - and therefore where the gaps continue to lie. In this paper, I found it very hard to identify what new knowledge or value this scoping review will produce. Thus, I think the authors need to make it much clearer how this piece of work will add to the existing evidence base.<br>2. The paramount concern in this study revolves around the lack of a clearly elucidated rationale for the gap in existing research. Further comprehensive literature is indispensable to substantiate the motivations and significance underlying the pursuit of this study.<br>3. The introduction of this article should place greater emphasis on an international perspective, as the readership is global. Furthermore, the title itself underscores its global focus. Please included more international references.<br>4. More references are needed for the 4th paragraph.<br>5. “However, it is unclear what information is available in literature about the support enabling nurses to develop as clinical academics.” This sentence could be revised as “There is no related literature review systematically…” to propose he research gap which could lead to research goal better.<br>6. “Scoping reviews have shown to be an essential tool to summarise all studies, in an accessible document, that could inform future actions.” In my opinion, this sentence doesn't effectively explain why the Scoping Review methodology was chosen to achieve the study's objectives.<br>(1) Munn, Z., Peters, M. D., Stern, C., Tufanaru, C., McArthur, A., & Aromataris, E. (2018). Systematic review or scoping review? Guidance for authors when choosing between a systematic or scoping review approach. BMC medical research methodology, 18, 1-7.<br>(2) 11.1.1 Why a scoping review? in JBI manual for evidence synthesis.<br>These two references may provide better explanatory statements for justifying your choice of the Scoping Review methodology.<br>7. The author mentioned, "It will also clarify and map key concepts and definitions underpinning the supervision required for nurses undertaking clinical academic development". The research questions and the research methodology, especially regarding data extraction, do not contain corresponding content.<br>8. The NHS in the first paragraph needs to have a full name.<br><br>METHODS:<br>1. to update the research methodology using the latest JBI methodology and cite the 2021 version as a reference. The methodology for Scoping Review in the latest JBI handbook also takes into consideration the method proposed by Arksey and O’Malley. |

| | |
|---|---|
| | Peters MDJ GC, McInerney P, Munn Z. Chapter 11: Scoping Reviews (2020 version). In: Aromataris E, Munn Z, eds. JBI Manual for Evidence Synthesis, 2020.<br>2. “Nursing is a profession which is regulated by law in the UK, and the Professional Qualifications Bill (UK Parliament, 2022) sets out that a profession is regulated where there is a legal requirement to have certain qualifications or experience to undertake certain professional activities or use a protected title (Department for Business Energy & Industrial Strategy, 2021)” This paragraph and the referenced UK perspective may not necessarily apply to other contexts. It is advisable to remove this description and provide a clearer definition of the population category. Population needs further refinement i.e. are you including all nursing professionals you have different forms of registrations? Ie. CNA, EN, RN? If there is no specific context then terms from other countries should be considered.<br>3. The scope of the “Concept” is not sufficiently clear and requires optimization. For example, the statement, "The focus will be on nurses who are aspiring to explore or pursue clinical academic development, or who already identify as a clinical academic," should be considered as inclusion criteria for the “Population”. However, it is currently placed under the "Concept" section.<br>Furthermore, should the focus exclusively be on “nurses who aspire to explore or pursue clinical academic development or those who already identify as clinical academics”? What if there are programs aimed at promoting clinical academic development for all nurses, not just those aspiring to it? Should they be included? Additionally, how can we objectively determine whether a nurse fits the criteria of being "aspiring to explore or pursue clinical academic development"?<br>4. The current inclusion and exclusion criteria based on the PCC (Population, Concept, and Context) framework are still not sufficiently clear, making it challenging to determine which studies will be included. To address this, we need to further clarify all components while maintaining consistency with the research questions. Based on the specific research question, we should provide a more precise description of the PCC elements and develop reasonable inclusion and exclusion criteria.<br>5. Why is the "context" limited to "any healthcare system in high-income countries"? The introduction section does not provide a rationale for this limitation. If such a limitation is imposed, please provide a valid reason and specify how "high-income countries" are defined.<br>6. The main research question mentioned “clinical academic opportunities”. This is not consistent with title, specific research questions (all are about programs), and the data extracted.<br>7. The literature retrieval strategy is simple, potentially leading to the omission of relevant literature. Furthermore, the current inclusion and exclusion criteria remain insufficiently clear and require further optimization based on the final criteria determined. The strategy for searching gray literature lacks specificity: for instance, which websites to search, and how to conduct these searches are not clearly defined.<br>8. According to JBI methodology, both literature screening and data extraction should undergo a piloting phase with two individuals before proceeding formally. Furthermore, formal literature screening and data extraction processes should involve the participation of two individuals throughout, rather than only partial involvement by two individuals. This is essential to mitigate substantial biases, |

| | |
|---|---|
| | particularly in the case of scoping reviews, which are characterized by greater flexibility and heterogeneity.<br>9. Patient and public involvement could be improved. Actually, this part does not just mean "Patient and public". I think you could understand it as "stakeholders' involvement". I think the involvement of stakeholders - nurses "who are aspiring to explore or pursue clinical academic development, or who already identify as a clinical academic" or nurse managers could improve the quality of this review. For example, they may tell you except programs, if there are other opportunities for this topic. I think this would make your research scope more clear and more appropriate. In literature search, they may provide more possible searching terms. For inclusion and exclusion criteria, they may provide more information. For the presentation of the results, they also could provide their perspectives which are important for this review. You could consider more.<br><br>DISCUSSION:<br>1. "The findings of this scoping review will inform future work with nurses to identify their awareness and experience of clinical academic careers, and to explore the lived experiences nurse researchers." This does not correspond to the research questions and the information to be extracted. |

| | |
|---|---|
| **REVIEWER NAME** | ***Hongli Sam Goh*** |
| **REVIEWER AFFILIATION** | National University of Singapore |
| **REVIEWER CONFLICT OF INTEREST** | No competing interest. |
| **DATE REVIEW RETURNED** | 07-Oct-2023 |

| | |
|---|---|
| **GENERAL COMMENTS** | Dear authors, thank you for the opportunity to review this protocol. The structure of this scoping review protocol is generally appropriate, detailed and aligned with the prescribed methodology.<br><br>May I suggest the following comments for the authors to further enhance their manuscript?<br>1) Clinical academic development: Define this concept.<br>2) State the period of literature search.<br>3) High-income countries: state rationale why they choose to focus on these countries.<br>4) Any consideration of non-English literature?<br>5) Is the referencing supposed to be written in APA format? If so, the end-referencing requires some editing to adhere to the format. E.g. "pp. 1-10" is incorrect. |

**VERSION 1 – AUTHOR RESPONSE**

| Reviewer 1 Comments | | Feedback | |
|---|---|---|---|
| Title | | | Line number reference |
| 1 | The word "globally" in the title is not appropriate for the following reasons: the database only includes a few | Many thanks for this suggestion – the databases utilised support international searching, and therefore not confined to | 2 |

| | | | |
|---|---|---|---|
| | English databases; the literature is limited to English-language sources; and the "context" section restricts its coverage to high-income countries. | identifying UK articles only. As the review is being undertaken as a PhD, we are limited to only including articles that are available in English language and we will acknowledge this as a limitation.<br><br>We have amended the protocol so that we do not exclude low-income countries. | |
| 2 | The term "mechanism" is not appropriate for the following reasons: based on the specific research question posed in the article, this review only focuses on relevant supporting programs and does not consider other supporting opportunities; it exclusively concentrates on "WHAT-programs" without addressing "HOW-how do these programs support clinical nurses' clinical academic development." Maybe “supporting programs” is more appropriate based on research questions you proposed. If you want to use “supporting programs” in the title, you need to define it clearly and make a summarized initial literature review on “supporting programs” in Introduction. Furthermore, you also need to use consist term in the whole paper, for examples, title, abstract, intro, method…… However, I found you also used “mechanism”, “supporting opportunities”, et al. I think these words have different.<br><br>scopes and definitions; this would make readers confused. KEEP CONSISTENTLY, This is important! | Many thanks for your feedback – the protocol reflects that we do not know how these clinical academic programmes support early career nurses, but we will be extracting this from the data. Terminology has been reviewed throughout the protocol to reflect consistency- thank you for this suggestion.<br><br>Comment: I still think “mechanism” is not appropriate for the title. I could not find any information about mechanism in the whole protocol. If you want to explore mechanism, you need to define it clearly in protocol and explain which information you will extract to provide mechanism information. If you are not clear, just use “programs” / “opportunities” in the title and the whole manuscript. The protocol should be a specific and final plan of the review, rather than a vague and preliminary draft of plan.<br><br>*Following further comments:*<br><br>The title has been amended – we have deleted the term mechanism and the protocol is now titled:<br><br>Opportunities for nurses aspiring to or undertaking clinical academic development globally – protocol for a scoping review. We think this makes the focus of this scoping review clearer; we hope that it will support work exploring mechanisms in future but acknowledge that this is beyond the scope of this review. | 1-2 |

| | | | |
|---|---|---|---|
| Abstract | | | |
| 1 | "We will include articles with a focus on nurses who are interested in undertaking clinical academic development, or a focus on programmes aimed at supporting clinical academic development for nurses." The sentence uses 'or' as a connecting word. This means that if the literature solely focuses on 'nurses who are interested in undertaking clinical academic development,' it could be included. However, it just means that the population criterion has been met. Nevertheless, I believe that the included literature should meet all the criteria of PCC. | Many thanks for this suggestion – we would like to identify both nurses who are undertaking clinical academic development or identify any clinical academic programmes that is aimed at supporting nurses. We hope that by covering both angles, we will identify all possible support being offered to nurses who are interested/pursuing clinical academic development.<br><br>Comment: However, you did not revise the related contents in the Abstract.<br><br>*Following further comments:*<br><br>Thanks for this; we have further reviewed and recognise that this was unintentionally confusing. Whilst papers focused on the programmes would meet our criteria they should not be considered as having an alternative focus. We have edited to make this inclusion statement fully aligned with the PCC. | 35-37 |
| 2 | "A proportion…" is not appropriate. The reason will be shown in the comments for Methods. | Many thanks – this has been reviewed and removed from protocol. | N/A |
| Strengths and limitations | | | |
| 1 | BMJ Open requires authors to write strengths and limitations of METHODS. The first one and the fourth on could not meet the criterion. | Many thanks – the 1st and 4th have been removed from this section<br><br>Comment: For the 3rd limitation, what limitation would be lead to because of "the only a proportion of the screening will be undertaken by a second reviewer" | 53 - 63 |

| | | | |
|---|---|---|---|
| | | *Following further comments:*<br><br>This is part of a PhD project being undertaken part time with limited resource and therefore we are taking a pragmatic approach to this, due to limited resources and an anticipated large volume of records to review. We have added the following text "This increases the likelihood of bias or error; this potential will be mitigated by use of a triage process to ensure a second reviewer considers all records where there is any uncertainty. " | |
| Introduction | | | |
| 1 | I would like to see a clearer and longer explication of what is already known - and therefore where the gaps continue to lie. In this paper, I found it very hard to identify what new knowledge or value this scoping review will produce. Thus, I think the authors need to make it much clearer how this piece of work will add to the existing evidence base | Thank you for this suggestion – we have reviewed the introduction section and we hope that the section now reflects how it will add to the evidence base.<br><br>Comment: The 1st and 2nd paragraphs could be combined as one paragraph.<br>Pg. 3 Line 101: c,all<br><br>*Following further comments:*<br><br>Many thanks – we have amended this as suggested. | 64 - 142 |
| 2 | The paramount concern in this study revolves around the lack of a clearly elucidated rationale for the gap in existing research. Further comprehensive literature is indispensable to substantiate the motivations and significance underlying the pursuit of this study. | Thank you – we have substantially amended the introduction to elucidate what literature is available for us to understand these clinical academic opportunities, which is why we are undertaking a scoping review to summarise and identify research gaps.<br><br>Comment: I think the introduction still did not provide information about existing related evidence on the topic. I understand the details of the related evidence will be shown in the results of this scoping review. However, if there are | 64 – 142 |

| | | | |
|---|---|---|---|
| | | no related original studies on the topic you interested in, conducting a scoping review is impossible. Therefore, except the significance of the topic (you have explained clearly in the introduction), give a brief literature review of the original studies on this topic from an international perspective is also important.<br><br>*Following further comments:* Thankyou for further sharing your thoughts on this – we have undertaken a rapid review highlighting that the area is underexplored and most of this in within the grey literature, hence a need for this scoping review to be undertaken. | <br><br><br><br><br><br><br><br>108-129 |
| 3 | The introduction of this article should place greater emphasis on an international perspective, as the readership is global. Furthermore, the title itself underscores its global focus. Please included more international references | Many thanks – this has been reviewed and international references have been included, highlighting a need to engage nurses in research. | 64 - 142 |
| 4 | More references are needed for the 4th paragraph. | Thank you for highlighting this – this has been reviewed and additional references have been included. | 89-98 |
| 5 | "However, it is unclear what information is available in literature about the support enabling nurses to develop as clinical academics." This sentence could be revised as "There is no related literature review systematically…" to propose he research gap which could lead to research goal better | Many thanks for this suggestion – this has now been amended | 111 |
| 6 | "Scoping reviews have shown to be an essential tool to summarise all studies, in an accessible document, that could inform future actions." In my opinion, this sentence doesn't effectively explain why the Scoping Review methodology was chosen to achieve the study's objectives.<br><br>(1) Munn, Z., Peters, M. D., Stern, C., Tufanaru, C., McArthur, A., & Aromataris, E. (2018). Systematic review or scoping review? Guidance | Thank you – we have reviewed this statement and added further explanation as to why a scoping review has been the chosen methodology. | 137 |

| | | | |
|---|---|---|---|
| | for authors when choosing between a systematic or scoping review approach. BMC medical research methodology, 18, 1-7.<br><br>(2) 11.1.1 Why a scoping review? in JBI manual for evidence synthesis.<br><br>These two references may provide better explanatory statements for justifying your choice of the Scoping Review methodology. | | |
| 7 | The author mentioned, "It will also clarify and map key concepts and definitions underpinning the supervision required for nurses undertaking clinical academic development". The research questions and the research methodology, especially regarding data extraction, do not contain corresponding content | Many thanks for highlighting this – we have reviewed and amended to reflect consistency<br><br>Comment: "What are the concepts described (type, duration, provider) by these clinical academic development programmes?" What are the concepts this study focuses on? Be specific. Furthermore, the concepts information is not shown in "Collating and summarising the results" part. Type, duration, provider, et al should be the information of the characteristics of the programs. The aims and characteristics should not be deleted.<br><br>***Following further comment:***<br><br>We have amended the 'Objectives' section to state *'clarify and map key concepts and definitions underpinning the <u>support</u> required for nurses undertaking clinical academic development..'*.<br><br>In the 'Identifying the research question section, we have reintroduced the sub-question reflecting the aims and characteristics of the programmes.<br><br>In the 'Collating and summarising the results' we have explicitly stated the key characteristics, which are directly reflected from the research questions. We have also added an explanation of what we mean but concepts in both the objectives and the questions to clarify | 145-150 |

| | | | |
|---|---|---|---|
| | | that we are concerned with how clinical academic development and opportunity are conceptualised in studies. We're grateful for this opportunity to clarify our meaning and appreciate your attention to language to enable this. | |
| 8 | The NHS in the first paragraph needs to have a full name. | Many thanks – this has been amended | 67 |
| Methods | | | |
| 1 | to update the research methodology using the latest JBI methodology and cite the 2021 version as a reference. The methodology for Scoping Review in the latest JBI handbook also takes into consideration the method proposed by Arksey and O'Malley. Peters MDJ GC, McInerney P, Munn Z. Chapter 11: Scoping Reviews (2020 version). In:<br><br>Aromataris E, Munn Z, eds. JBI Manual for Evidence Synthesis, 2020. | Many thanks for this suggestion – the reference has been updated | 152 |
| 2 | "Nursing is a profession which is regulated by law in the UK, and the Professional Qualifications Bill (UK Parliament, 2022) sets out that a profession is regulated where there is a legal requirement to have certain qualifications or experience to undertake certain professional activities or use a protected title (Department for Business Energy & Industrial Strategy, 2021)" This paragraph and the referenced UK perspective may not necessarily apply to other contexts. It is advisable to remove this description and provide a clearer definition of the population category. Population needs further refinement i.e. are you including all nursing professionals you have different forms of registrations? Ie. CNA, EN, RN? If there is no specific context then terms from other countries should be considered. | Many thanks for this suggestion – amendments have been made to the protocol to reflect inclusion of all registered nursing professionals worldwide. Within the Population, we have deleted references that would exclude different forms of registration, and there is explicitly no country limits.<br><br>Comment: I did not find revisions in the revised version of manuscript. (Population/types of participants in Identifying the research question).<br><br>***Following further comment:***<br><br>We have noted within this section that, as registration may vary in different countries worldwide, we are including all registered professionals. We have noted how nurses within the UK are registered, but we do not feel it is feasible to | 167-173 |

| | | | |
|---|---|---|---|
| | | document how registration may differ in every country.<br><br>We have edited to clarify – here and in the inclusion criteria – that we will accept and include those described or considered as registered nurses. | |
| 3 | The scope of the “Concept” is not sufficiently clear and requires optimization. For example, the statement, "The focus will be on nurses who are aspiring to explore or pursue clinical academic development, or who already identify as a clinical academic," should be considered as inclusion criteria for the “Population”. However, it is currently placed under the "Concept" section. Furthermore, should the focus exclusively be on “nurses who aspire to explore or pursue clinical academic development or those who already identify as clinical academics”? What if there are programs aimed at promoting clinical academic development for all nurses, not just those aspiring to it? Should they be included? Additionally, how can we objectively determine whether a nurse fits the criteria of being "aspiring to explore or pursue clinical academic development"? | Many thanks for the suggestion – we felt that it was important to define the search terms explicitly alongside the PCC definition, which is why we have included registered nurses as the population.<br><br>The concept has been reviewed to reflect that we are aiming to identify those within the population who are undertaking clinical academic development, and also the programmes that could be undertaken by them. We agree with your statement that we cannot objectively determine if a nurse fits the criteria of being "aspiring to explore or pursue clinical academic development". However, we hope that this scoping review will identify programmes of work that are not just for those in established clinical academic programmes, such as ‘taster’ courses or workshops and this concept is written to capture this; we have amended to make this clearer.<br><br>Comment: I did not find revisions based on the comments in the<br><br>revised version of manuscript. (Concept in Identifying the<br><br>research question).<br><br>For example, the focus will be on nurses who are aspiring to<br><br>explore or pursue clinical academic development, or who already<br><br>identify as a clinical academic," should be considered as inclusion<br><br>criteria for the “Population”. However, it is currently placed | N/A |

| | | | |
|---|---|---|---|
| | | under the "Concept" section. This should be removed to<br><br>“Population/types of participants in Identifying the research<br><br>question” part.<br><br><br>“Furthermore, should the focus exclusively be on “nurses wh”o<br><br>aspire to explore or pursue clinical academic development or<br><br>those who already identify as clinical academics”? What if there<br><br>are programs aimed at promoting clinical academic development<br><br>for all nurses, not just those aspiring to it? Should they be<br><br>included?” This comment is also not be solved.<br><br><br>Meanwhile, I still think you should give clear criteria for "aspiring<br><br>to explore or pursue clinical academic development", otherwise<br><br>the inclusion criteria for literature would not be clear.<br><br><br>***Following further comment:***<br><br>Thank you for your continued thoughtful engagement with this. We really appreciate your help in articulating this non-simple concept. We have edited to clarify that we are including a very broad approach to this early stage of clinical academic exploration, including programmes which are aimed at fostering such exploration and aspiration. We have edited substantively in order to better capture our meaning. We have | |

| | | | |
|---|---|---|---|
| | | also edited our inclusion criteria to further capture this. | |
| 4 | The current inclusion and exclusion criteria based on the PCC (Population, Concept, and Context) framework are still not sufficiently clear, making it challenging to determine which studies will be included. To address this, we need to further clarify all components while maintaining consistency with the research questions. Based on the specific research question, we should provide a more precise description of the PCC elements and develop reasonable inclusion and exclusion criteria. | With extensive support from healthcare librarians (hospital and university), we have tested several terms within each domain and within each database. Due to the number of results received and on reviewing the literature obtained, we have deemed it appropriate to exclude several terms as they do not offer anything further to the scope.<br><br>For example, in Medline, for Concept, we have tested in title or abstract or keyword:<br><br>• Clinical academi*<br>• Research* training<br>• Research intern*<br>• Research career*<br>However, by adding the term ‘Fellow*’ suggested a medical focus and therefore we have not included this.<br><br>Full transparency of the search terms suggested with the healthcare librarians has been uploaded for further review.<br><br>Comment: I did not find revisions based on the left comments in the revised version of manuscript. (PCC in Identifying the research question). The clear definition of PCC is very important for this protocol. However, I did not find further clarification based on the comments in the revised version.<br><br>***Following further comment:***<br><br>*Thanks for this. We have set out our inclusion and exclusion criteria more clearly and fully to explain in detail how we will apply the PCC (which we have further edited in response to previous comments.* We have uploaded the search strategy and clarified this is available as supplementary material, and | 188 |

| | | | |
|---|---|---|---|
| | | we have now added in a box, sharing the terms searched for each concept. | |
| 5 | Why is the "context" limited to "any healthcare system in high-income countries"? The introduction section does not provide a rationale for this limitation. If such a limitation is imposed, please provide a valid reason and specify how "high-income countries" are defined. | Many thanks for your feedback – the context has been amended to reflect international context without limiting to countries based on income. | 186 |
| 6 | The main research question mentioned “clinical academic opportunities”. This is not consistent with title, specific research questions (all are about programs), and the data extracted. | Many thanks – this has now been reviewed throughout the protocol to reflect more consistent terminology<br><br>Comment: However, you still use “opportunities” here and “programs” in other places. What are the differences between them? Or they are the same? However, I think opportunities include more than programs.<br><br>***Following further comment:***<br><br>Many thanks for your feedback – we have reviewed each mention of ‘opportunities’ and ‘programmes’ throughout the manuscript. We have now amended this. | N/A |
| 7 | The literature retrieval strategy is simple, potentially leading to the omission of relevant literature. Furthermore, the current inclusion and exclusion criteria remain insufficiently clear and require further optimization based on the final criteria determined. The strategy for searching gray literature lacks specificity: for instance, which websites to search, and how to conduct these searches are not clearly defined | The literature retrieval strategy has been uploaded and has been extensively reviewed and tested with healthcare librarians.<br><br>In relation to the grey literature, a targeted approach using information from local, regional and national clinical academic networks.<br><br>Comment: In the protocol, the websites and searching method for grey literature should be determined. Give specific information in the manuscript. | |

| | | | |
|---|---|---|---|
| | | ***Following further comment:***<br><br>We have noted within this section that we will be identifying organisations through NIHR and HEE. Additionally, we will be inviting our clinical academic networks to recommend organisations, and this is now stated within the PPI section. The details of the search will be shared when the scoping review is undertaken. | |
| 8 | According to JBI methodology, both literature screening and data extraction should undergo a piloting phase with two individuals before proceeding formally. Furthermore, formal literature screening and data extraction processes should involve the participation of two individuals throughout, rather than only partial involvement by two individuals. This is essential to mitigate substantial biases, particularly in the case of scoping reviews, which are characterized by greater flexibility and heterogeneity | This is part of a PhD project being undertaken part time with limited resource and therefore we are taking a pragmatic approach to this, due to limited resources and an anticipated large volume of records to review. Following initial piloting on a small subset of records by two independent reviewers, one reviewer will screen the titles and abstracts and a minimum of 30% of records will be independently screened by the second reviewer. Any records identified as being of uncertain relevance, or sharing characteristics with such records will also be reviewed by the second reviewer or a third reviewer. This “triage” process will ensure that all records for which there is any uncertainty over relevance will be double screened as a minimum.<br><br>Comment: This clarification should be provided in the protocol.<br><br>***Following further comment:***<br><br>Many thanks – this has now been included and we hope this answers your comment. | 276-288 |
| 9 | Patient and public involvement could be improved. Actually, this part does not just mean “Patient and public”. I think you could understand it as “stakeholders’ involvement”. I think the involvement of stakeholders - nurses “who are aspiring to explore or pursue clinical academic development, or who already identify as a clinical academic” | Many thanks for this suggestion – stakeholder involvement and engagement will be invited by sharing the protocol and presentation of the review with our clinical academic networks. | 346-352 |

| | | | |
|---|---|---|---|
| | or nurse managers could improve the quality of this review. For example, they may tell you except programs, if there are other opportunities for this topic. I think this would make your research scope more clear and more appropriate. In literature search, they may provide more possible searching terms. For inclusion and exclusion criteria, they may provide more information. For the presentation of the results, they also could provide their perspectives which are important for this review. You could consider more. | Comment: This part should provide more information about how the stakeholders being involved in the review/study.<br><br>***Following further comment:***<br><br>The editorial comments have noted that as we not engaged with patient and public involvement, we can document 'None'. However, following the comment you have originally raised, we have revised this to reflect stakeholder involvement, and explicitly shared which areas we would like them to review. | |
| Discussion | | | |
| 1 | "The findings of this scoping review will inform future work with nurses to identify their awareness and experience of clinical academic careers, and to explore the lived experiences nurse researchers. " This does not correspond to the research questions and the information to be extracted | Many thanks for your feedback – this had been reviewed and amended. Following editorial comments, this section has now been removed as a discussion section is optional for protocol manuscript. | N/A |
| | **Reviewer 2 Comments** | **Feedback** | |
| 1 | Clinical academic development: Define this concept. | Many thanks for this feedback – the introduction section has been reviewed and we feel that we have addressed this comment within the section. | 63 - 142 |
| 2 | State the period of literature search. | We recognise that clinical academic development has only become prominent in the last 20 years. However, we wanted to ensure inclusion of any earlier seminal work. Therefore, we have noted that the databases will be searched from database inception. | 213 |
| 3 | High-income countries: state rationale why they choose to focus on these countries. | Many thanks for noting this – following major revisions, we have deleted this from the protocol | N/A |
| 4 | Any consideration of non-English literature? | We have noted that, due to lack of funding for translation services, we are unable to include those that are not available in English language. | 265 |
| 5 | Is the referencing supposed to be written in APA format? If so, the end- | This has been reviewed accordingly – the referencing is now in Vancouver | 404 |

| | | | |
|---|---|---|---|
| | referencing requires some editing to adhere to the format. E.g. "pp. 1-10" is incorrect. | format and the end-referencing has been edited accordingly. | |

**VERSION 2 – REVIEW**

| | |
|---|---|
| **REVIEWER NAME** | ***Qirong Chen*** |
| **REVIEWER AFFILIATION** | Central South University, Xiangya School of Nursing |
| **REVIEWER CONFLICT OF INTEREST** | No competing interest. |
| **DATE REVIEW RETURNED** | 15-Jan-2024 |

| | |
|---|---|
| **GENERAL COMMENTS** | See the comments below. Most comments still need to be handled. |

| Title | | | Line number reference |
|---|---|---|---|
| 2 | The term "mechanism" is not appropriate for the following reasons: based on the specific research question posed in the article, this review only focuses on relevant supporting programs and does not consider other supporting opportunities; it exclusively concentrates on "WHAT-programs" without addressing "HOW-how do these programs support clinical nurses' clinical academic development." Maybe “supporting programs” is more appropriate based on research questions you proposed. If you want to use “supporting programs” in the title, you need to define it clearly and make a summarized initial literature review on “supporting programs” in Introduction. Furthermore, you also need to use consist term in the whole paper, for examples, title, abstract, intro, method…… However, I found you also used “mechanism”, “supporting opportunities”, et al. I think these words have different scopes and definitions; this would make readers confused. KEEP CONSISTENTLY, This is important! | Many thanks for your feedback – the protocol reflects that we do not know how these clinical academic programmes support early career nurses, but we will be extracting this from the data. Terminology has been reviewed throughout the protocol to reflect consistency- thank you for this suggestion.<br><br>Comment: I still think “mechanism” is not appropriate for the title. I could not find any information about mechanism in the whole protocol. If you want to explore mechanism, you need to define it clearly in protocol and explain which information you will extract to provide mechanism information. If you are not clear, just use “programs” / “opportunities” in the title and the whole manuscript. The protocol should be a specific and final plan of the review, rather than a vague and preliminary draft of plan. | N/A |
| Abstract | | | |

| | | | |
|---|---|---|---|
| 1 | "We will include articles with a focus on nurses who are interested in undertaking clinical academic development, or a focus on programmes aimed at supporting clinical academic development for nurses." The sentence uses 'or' as a connecting word. This means that if the literature solely focuses on 'nurses who are interested in undertaking clinical academic development,' it could be included. However, it just means that the population criterion has been met. Nevertheless, I believe that the included literature should meet all the criteria of PCC. | Many thanks for this suggestion – we would like to identify both nurses who are undertaking clinical academic development and identify any clinical academic programmes that is aimed at supporting nurses. We hope that by covering both angles, we will identify all possible support being offered to nurses who are interested/pursuing clinical academic development.<br><br>Comment: However, you did not revise the related contents in the Abstract. | N/A |
| Strengths and limitations | | | |
| 1 | BMJ Open requires authors to write strengths and limitations of METHODS. The first one and the fourth on could not meet the criterion. | Many thanks – the 1st and 4th have been removed from this section<br><br>Comment: For the 3rd limitation, what limitation would be lead to because of "the only a proportion of the screening will be undertaken by a second reviewer". | 55 + 62 |
| Introduction | | | |
| 1 | I would like to see a clearer and longer explication of what is already known - and therefore where the gaps continue to lie. In this paper, I found it very hard to identify what new knowledge or value this scoping review will produce. Thus, I think the authors need to make it much clearer how this piece of work will add to the existing evidence base | Thank you for this suggestion – we have reviewed the introduction section and we hope that the section now reflects how it will add to the evidence base.<br><br>Comment: The 1st and 2nd paragraphs could be combined as one paragraph.<br><br>Pg. 3 Line 101: c,all | 71 - 129 |
| 2 | The paramount concern in this study revolves around the lack of a clearly elucidated rationale for the gap in existing research. Further comprehensive literature is indispensable to substantiate the | Thank you – we have substantially amended the introduction to elucidate what literature is available for us to understand these clinical academic opportunities, which is why we are | 125 |

| | | | |
|---|---|---|---|
| | motivations and significance underlying the pursuit of this study. | undertaking a scoping review to summarise and identify research gaps.<br><br>Comment: I think the introduction still did not provide information about existing related evidence on the topic. I understand the details of the related evidence will be shown in the results of this scoping review. However, if there are no related original studies on the topic you interested in, conducting a scoping review is impossible. Therefore, except the significance of the topic (you have explained clearly in the introduction), give a brief literature review of the original studies on this topic from an international perspective is also important. | |
| 7 | The author mentioned, "It will also clarify and map key concepts and definitions underpinning the supervision required for nurses undertaking clinical academic development". The research questions and the research methodology, especially regarding data extraction, do not contain corresponding content | Many thanks for highlighting this – we have reviewed and amended to reflect consistency<br><br>Comment: “What are the concepts described (type, duration, provider) by these clinical academic development programmes?”<br><br>What are the concepts this study focuses on? Be specific.<br><br>Furthermore, the concepts information is not shown in “Collating and summarising the results” part. Type, duration, provider, et al should be the information of the characteristics of the programs. The aims and characteristics should not be deleted. | 174 |
| Methods | | | |
| 2 | “Nursing is a profession which is regulated by law in the UK, and the Professional Qualifications Bill (UK Parliament, 2022) sets out that a profession is regulated where there is a legal requirement to have certain qualifications or experience to undertake certain professional | Many thanks for this suggestion – amendments have been made to the protocol to reflect inclusion of all registered nursing professionals worldwide. Within the Population, we have deleted references that would exclude different forms of registration, and there is explicitly no country limits. | 147 |

| | | | |
|---|---|---|---|
| | activities or use a protected title (Department for Business Energy & Industrial Strategy, 2021)" This paragraph and the referenced UK perspective may not necessarily apply to other contexts. It is advisable to remove this description and provide a clearer definition of the population category. Population needs further refinement i.e. are you including all nursing professionals you have different forms of registrations? Ie. CNA, EN, RN? If there is no specific context then terms from other countries should be considered. | Comment: I did not find revisions in the revised version of manuscript. (Population/types of participants in Identifying the research question). | |
| 3 | The scope of the "Concept" is not sufficiently clear and requires optimization. For example, the statement, "The focus will be on nurses who are aspiring to explore or pursue clinical academic development, or who already identify as a clinical academic," should be considered as inclusion criteria for the "Population". However, it is currently placed under the "Concept" section. Furthermore, should the focus exclusively be on "nurses who aspire to explore or pursue clinical academic development or those who already identify as clinical academics"? What if there are programs aimed at promoting clinical academic development for all nurses, not just those aspiring to it? Should they be included? Additionally, how can we objectively determine whether a nurse fits the criteria of being "aspiring to explore or pursue clinical academic development"? | Many thanks for the suggestion – we felt that it was important to define the search terms explicitly alongside the PCC definition, which is why we have included registered nurses as the population.<br><br>The concept has been reviewed to reflect that we are aiming to identify those within the population who are undertaking clinical academic development, and also the programmes that could be undertaken by them. We agree with your statement that we cannot objectively determine if a nurse fits the criteria of being "aspiring to explore or pursue clinical academic development". However, we hope that this scoping review will identify programmes of work that are not just for those in established clinical academic programmes, such as 'taster' courses or workshops and this concept is written to capture this; we have amended to make this clearer.<br><br>Comment: I did not find revisions based on the comments in the revised version of manuscript. (Concept in Identifying the research question).<br><br>For example, the focus will be on nurses who are aspiring to explore or pursue clinical academic development, or who already identify as a clinical academic," should be considered as inclusion criteria for the "Population". However, it is | |

| | | | |
|---|---|---|---|
| | | currently placed under the "Concept" section. This should be removed to “Population/types of participants in Identifying the research question” part.<br><br>“Furthermore, should the focus exclusively be on “nurses wh”o aspire to explore or pursue clinical academic development or those who already identify as clinical academics”? What if there are programs aimed at promoting clinical academic development for all nurses, not just those aspiring to it? Should they be included?” This comment is also not be solved.<br><br>Meanwhile, I still think you should give clear criteria for "aspiring to explore or pursue clinical academic development", otherwise the inclusion criteria for literature would not be clear. | |
| 4 | The current inclusion and exclusion criteria based on the PCC (Population, Concept, and Context) framework are still not sufficiently clear, making it challenging to determine which studies will be<br><br>included. To address this, we need to further clarify all components while maintaining consistency with the research questions. Based on the specific research question, we should provide a more<br><br>precise description of the PCC elements and develop reasonable inclusion and exclusion criteria. | With extensive support from healthcare librarians (hospital and university), we have tested several terms within each domain and within each database. Due to the number of results received and on reviewing the literature obtained, we have deemed it appropriate to exclude several terms as they do not offer anything further to the scope.<br><br>For example, in Medline, for Concept, we have tested in title or abstract or keyword:<br>• Clinical academi*<br>• Research* training<br>• Research intern*<br>• Research career*<br>However, by adding the term ‘Fellow*’ suggested a medical focus and therefore we have not included this.<br><br>Full transparency of the search terms suggested with the healthcare librarians has been uploaded for further review. | N/A |

| | | | |
|---|---|---|---|
| | | Comment: I did not find revisions based on the left comments in the revised version of manuscript. (PCC in Identifying the research question). The clear definition of PCC is very important for this protocol. However, I did not find further clarification based on the comments in the revised version. | |
| 5 | Why is the "context" limited to "any healthcare system in high-income countries"? The introduction section does not provide a rationale for this limitation. If such a limitation is imposed, please provide a valid reason and specify how "high-income countries" are defined. | Many thanks for your feedback – the context has been amended to reflect international context without limiting to countries based on income. | 167 |
| 6 | The main research question mentioned “clinical academic opportunities”. This is not consistent with title, specific research questions (all are about programs), and the data extracted. | Many thanks – this has now been reviewed throughout the protocol to reflect more consistent terminology<br><br>Comment: However, you still use “opportunities” here and “programs” in other places. What are the differences between them? Or they are the same? However, I think opportunities include more than programs. | N/A |
| 7 | The literature retrieval strategy is simple, potentially leading to the omission of relevant literature. Furthermore, the current inclusion and exclusion criteria remain insufficiently clear and require further optimization based on the final criteria determined. The strategy for searching gray literature lacks specificity: for instance, which websites to search, and how to conduct these searches are not clearly defined | The literature retrieval strategy has been uploaded and has been extensively reviewed and tested with healthcare librarians.<br><br>In relation to the grey literature, a targeted approach using information from local, regional and national clinical academic networks.<br><br>Comment: In the protocol, the websites and searching method for grey literature should be determined. Give specific information in the manuscript. | |

| | | | |
|---|---|---|---|
| 8 | According to JBI methodology, both literature screening and data extraction should undergo a piloting phase with two individuals before proceeding formally. Furthermore, formal literature screening and data extraction processes should involve the participation of two individuals<br><br>throughout, rather than only partial involvement by two individuals. This is essential to mitigate substantial biases, particularly in the case of scoping reviews, which are characterized by greater flexibility and heterogeneity | This is part of a PhD project being undertaken part time with limited resource and therefore we are taking a pragmatic approach to this, due to limited resources and an anticipated large volume of records to review. Following initial piloting on a small subset of records by two independent reviewers, one reviewer will screen the titles and abstracts and a minimum of 30% of records will be independently screened by the second reviewer. Any records identified as being of uncertain relevance, or sharing characteristics with such records will also be reviewed by the second reviewer or a third reviewer. This “triage” process will ensure that all records for which there is any uncertainty over relevance will be double screened as a minimum.<br><br>Comment: This clarification should be provided in the protocol. | 233 |
| 9 | Patient and public involvement could be improved. Actually, this part does not just mean “Patient and public”. I think you could understand it as “stakeholders’ involvement”. I think the involvement of stakeholders - nurses “who are aspiring to explore or pursue clinical academic development, or who already identify as a clinical academic” or nurse managers could improve the quality of this review. For example, they may tell you except programs, if there are other opportunities for this<br><br>topic. I think this would make your research scope more clear and more appropriate. In literature search, they may provide more possible searching terms. For inclusion and exclusion criteria, they may provide more information. For the presentation of the results, they also could provide their perspectives which are important for this review. You could consider more. | Many thanks for this suggestion – stakeholder involvement and engagement will be invited by sharing the protocol and presentation of the review with our clinical academic networks<br><br>Comment: This part should provide more information about how the stakeholders being involved in the review/study. | 305 |
| Discussion | | | |

| | | | |
|---|---|---|---|
| 1 | “The findings of this scoping review will inform future work with nurses to identify their awareness and experience of clinical academic careers, and to explore the lived experiences nurse researchers. ” This does not correspond to the research questions and the information to be extracted | Many thanks for your feedback – this has been reviewed and amended | 309 |

**VERSION 2 – AUTHOR RESPONSE**

| Title | | | Line number reference |
|---|---|---|---|
| 1 | The word "globally" in the title is not appropriate for the following reasons: the database only includes a few English databases; the literature is limited to English-language sources; and the "context" section restricts its coverage to high-income countries. | Many thanks for this suggestion – the databases utilised support international searching, and therefore not confined to identifying UK articles only. As the review is being undertaken as a PhD, we are limited to only including articles that are available in English language and we will acknowledge this as a limitation.<br><br>The protocol will be amended so that we do not exclude low-income countries. | 3 |
| 2 | The term "mechanism" is not appropriate for the following reasons: based on the specific research question posed in the article, this review only focuses on relevant supporting programs and does not consider other supporting opportunities; it exclusively concentrates on "WHAT-programs" without addressing "HOW-how do these programs support clinical nurses' clinical academic development." Maybe “supporting programs” is more appropriate based on research questions you proposed. If you want to use “supporting programs” in the title, you need to define it clearly and make a summarized initial literature review on “supporting programs” in Introduction. Furthermore, you also need to use consist term in the whole paper, for examples, title, abstract, intro, method…… However, I found you also used “mechanism”, | Many thanks for your feedback – the protocol reflects that we do not know how these clinical academic programmes support early career nurses, but we will be extracting this from the data. Terminology has been reviewed throughout the protocol to reflect consistency- thank you for this suggestion. | N/A |

| | | | |
|---|---|---|---|
| | "supporting opportunities", et al. I think these words have different<br><br>scopes and definitions; this would make readers confused. KEEP CONSISTENTLY, This is important! | | |
| Abstract | | | |
| 1 | "We will include articles with a focus on nurses who are interested in undertaking clinical academic development, or a focus on programmes aimed at supporting clinical academic development for nurses." The sentence uses 'or' as a connecting word. This means that if the<br><br>literature solely focuses on 'nurses who are interested in undertaking clinical academic development,' it could be included. However, it just means that the population criterion has been met. Nevertheless, I believe that the included literature should meet all the criteria of PCC. | Many thanks for this suggestion – we would like to identify both nurses who are undertaking clinical academic development and identify any clinical academic programmes that is aimed at supporting nurses. We hope that by covering both angles, we will identify all possible support being offered to nurses who are interested/pursuing clinical academic development. | N/A |
| 2 | "A proportion…" is not appropriate. The reason will be shown in the comments for Methods. | Many thanks – this has been reviewed and removed from protocol. | 40 |
| Strengths and limitations | | | |
| 1 | BMJ Open requires authors to write strengths and limitations of METHODS. The first one and the fourth on could not meet the criterion. | Many thanks – the 1st and 4th have been removed from this section | 55 + 62 |
| Introduction | | | |
| 1 | I would like to see a clearer and longer explication of what is already known - and therefore where the gaps continue to lie. In this paper, I found it very hard to identify what new knowledge or value this scoping review will produce. Thus, I think the authors need to make it much clearer how this piece of work will add to the existing evidence base | Thank you for this suggestion – we have reviewed the introduction section and we hope that the section now reflects how it will add to the evidence base. | 71 - 129 |
| 2 | The paramount concern in this study revolves around the lack of a clearly elucidated rationale for the gap in existing research. Further | Thank you – we have substantially amended the introduction to elucidate what literature is available for us to understand these clinical academic | 125 |

| | | | |
|---|---|---|---|
| | comprehensive literature is indispensable to substantiate the motivations and significance underlying the pursuit of this study. | opportunities, which is why we are undertaking a scoping review to summarise and identify research gaps. | |
| 3 | The introduction of this article should place greater emphasis on an international perspective, as the readership is global. Furthermore, the title itself underscores its global focus. Please included more international references | Many thanks – this has been reviewed and international references have been included, highlighting a need to engage nurses in research. | 71 - 129 |
| 4 | More references are needed for the 4th paragraph. | Thank you for highlighting this – this has been reviewed and additional references have been included. | 110 and 113 |
| 5 | "However, it is unclear what information is available in literature about the support enabling nurses to develop as clinical academics." This sentence could be revised as "There is no related literature review systematically…" to propose he research gap which could lead to research goal better | Many thanks for this suggestion – this has now been amended | 118 |
| 6 | "Scoping reviews have shown to be an essential tool to summarise all studies, in an accessible document, that could inform future actions." In my opinion, this sentence doesn't effectively explain why the Scoping Review methodology was chosen to achieve the study's objectives.<br><br>(1) Munn, Z., Peters, M. D., Stern, C., Tufanaru, C., McArthur, A., & Aromataris, E. (2018). Systematic review or scoping review? Guidance for authors when choosing between a systematic or scoping review approach. BMC medical research methodology, 18, 1-7.<br><br>(2) 11.1.1 Why a scoping review? in JBI manual for evidence synthesis.<br><br>These two references may provide better explanatory statements for justifying your choice of the Scoping Review methodology. | Thank you – we have reviewed this statement and added further explanation as to why a scoping review has been the chosen methodology. | 125 |

| | | | |
|---|---|---|---|
| 7 | The author mentioned, "It will also clarify and map key concepts and definitions underpinning the supervision required for nurses undertaking clinical academic development". The research questions and the research methodology, especially regarding data extraction, do not contain corresponding content | Many thanks for highlighting this – we have reviewed and amended to reflect consistency | 174 |
| 8 | The NHS in the first paragraph needs to have a full name. | Many thanks – this has been amended | 72 |
| Methods | | | |
| 1 | to update the research methodology using the latest JBI methodology and cite the 2021 version as a reference. The methodology for Scoping Review in the latest JBI handbook also takes into consideration the method proposed by Arksey and O'Malley. Peters MDJ GC, McInerney P, Munn Z. Chapter 11: Scoping Reviews (2020 version). In:<br><br>Aromataris E, Munn Z, eds. JBI Manual for Evidence Synthesis, 2020. | Many thanks for this suggestion – the reference has been updated | 139 |
| 2 | "Nursing is a profession which is regulated by law in the UK, and the Professional Qualifications Bill (UK Parliament, 2022) sets out that a profession is regulated where there is a legal requirement to have certain qualifications or experience to undertake certain professional activities or use a protected title (Department for Business Energy & Industrial Strategy, 2021)" This paragraph and the referenced UK perspective may not necessarily apply to other contexts. It is advisable to remove this description and provide a clearer definition of the population category. Population needs further refinement i.e. are you including all nursing professionals you have different forms of registrations? Ie. CNA, EN, RN? If there is no specific context then terms from other countries should be considered. | Many thanks for this suggestion – amendments have been made to the protocol to reflect inclusion of all registered nursing professionals worldwide. Within the Population, we have deleted references that would exclude different forms of registration, and there is explicitly no country limits. | 147 |

| | | | |
|---|---|---|---|
| 3 | The scope of the “Concept” is not sufficiently clear and requires optimization. For example, the statement, "The focus will be on nurses who are aspiring to explore or pursue clinical academic development, or who already identify as a clinical academic," should be considered as inclusion criteria for the “Population”. However, it is currently placed under the "Concept" section. Furthermore, should the focus exclusively be on “nurses who aspire to explore or pursue clinical academic development or those who already identify as clinical academics”? What if there are programs aimed at promoting clinical academic development for all nurses, not just those aspiring to it? Should they be included? Additionally, how can we objectively determine whether a nurse fits the criteria of being "aspiring to explore or pursue clinical academic development"? | Many thanks for the suggestion – we felt that it was important to define the search terms explicitly alongside the PCC definition, which is why we have included registered nurses as the population.<br><br>The concept has been reviewed to reflect that we are aiming to identify those within the population who are undertaking clinical academic development, and also the programmes that could be undertaken by them. We agree with your statement that we cannot objectively determine if a nurse fits the criteria of being "aspiring to explore or pursue clinical academic development". However, we hope that this scoping review will identify programmes of work that are not just for those in established clinical academic programmes, such as ‘taster’ courses or workshops and this concept is written to capture this; we have amended to make this clearer. | |
| 4 | The current inclusion and exclusion criteria based on the PCC (Population, Concept, and Context) framework are still not sufficiently clear, making it challenging to determine which studies will be<br><br>included. To address this, we need to further clarify all components while maintaining consistency with the research questions. Based on the specific research question, we should provide a more<br><br>precise description of the PCC elements and develop reasonable inclusion and exclusion criteria. | With extensive support from healthcare librarians (hospital and university), we have tested several terms within each domain and within each database. Due to the number of results received and on reviewing the literature obtained, we have deemed it appropriate to exclude several terms as they do not offer anything further to the scope.<br><br>For example, in Medline, for Concept, we have tested in title or abstract or keyword:<br><br>• Clinical academi*<br>• Research* training<br>• Research intern*<br>• Research career*<br>However, by adding the term ‘Fellow*’ suggested a medical focus and therefore we have not included this.<br><br>Full transparency of the search terms suggested with the healthcare librarians has been uploaded for further review. | N/A |
| 5 | Why is the "context" limited to "any healthcare system in high-income countries"? The introduction section does not provide a rationale for this | Many thanks for your feedback – the context has been amended to reflect | 167 |

| | | | |
|---|---|---|---|
| | limitation. If such a limitation is imposed, please provide a valid reason and specify how "high-income countries" are defined. | international context without limiting to countries based on income. | |
| 6 | The main research question mentioned “clinical academic opportunities”. This is not consistent with title, specific research questions (all are about programs), and the data extracted. | Many thanks – this has now been reviewed throughout the protocol to reflect more consistent terminology | N/A |
| 7 | The literature retrieval strategy is simple, potentially leading to the omission of relevant literature. Furthermore, the current inclusion and exclusion criteria remain insufficiently clear and require further optimization based on the final criteria determined. The strategy for searching gray literature lacks specificity: for instance, which websites to search, and how to conduct these searches are not clearly defined | The literature retrieval strategy has been uploaded and has been extensively reviewed and tested with healthcare librarians.<br><br>In relation to the grey literature, a targeted approach using information from local, regional and national clinical academic networks. | |
| 8 | According to JBI methodology, both literature screening and data extraction should undergo a piloting phase with two individuals before proceeding formally. Furthermore, formal literature screening and data extraction processes should involve the participation of two individuals<br><br>throughout, rather than only partial involvement by two individuals. This is essential to mitigate substantial biases, particularly in the case of scoping reviews, which are characterized by greater flexibility and heterogeneity | This is part of a PhD project being undertaken part time with limited resource and therefore we are taking a pragmatic approach to this, due to limited resources and an anticipated large volume of records to review. Following initial piloting on a small subset of records by two independent reviewers, one reviewer will screen the titles and abstracts and a minimum of 30% of records will be independently screened by the second reviewer. Any records identified as being of uncertain relevance, or sharing characteristics with such records will also be reviewed by the second reviewer or a third reviewer. This “triage” process will ensure that all records for which there is any uncertainty over relevance will be double screened as a minimum. | 233 |
| 9 | Patient and public involvement could be improved. Actually, this part does not just mean “Patient and public”. I think you could understand it as “stakeholders’ involvement”. I think the involvement of stakeholders - nurses | Many thanks for this suggestion – stakeholder involvement and engagement will be invited by sharing the protocol and presentation of the review with our clinical academic networks | 305 |

| | | | |
|---|---|---|---|
| | "who are aspiring to explore or pursue clinical academic development, or who already identify as a clinical academic" or nurse managers could improve the quality of this review. For example, they may tell you except programs, if there are other opportunities for this topic. I think this would make your research scope more clear and more appropriate. In literature search, they may provide more possible searching terms. For inclusion and exclusion criteria, they may provide more information. For the presentation of the results, they also could provide their perspectives which are important for this review. You could consider more. | | |
| Discussion | | | |
| 1 | "The findings of this scoping review will inform future work with nurses to identify their awareness and experience of clinical academic careers, and to explore the lived experiences nurse researchers. " This does not correspond to the research questions and the information to be extracted | Many thanks for your feedback – this has been reviewed and amended | 309 |
